# Whole-genome transcription and DNA methylation analysis of peripheral blood mononuclear cells identified aberrant gene regulation pathways in systemic lupus erythematosus

**DOI:** 10.1186/s13075-016-1050-x

**Published:** 2016-07-13

**Authors:** Honglin Zhu, Wentao Mi, Hui Luo, Tao Chen, Shengxi Liu, Indu Raman, Xiaoxia Zuo, Quan-Zhen Li

**Affiliations:** Department of Rheumatology, Xiangya Hospital, Central South University, 87 Xiangya Road, Changsha, Hunan, 410008 People’s Republic of China; Department of Immunology, University of Texas Southwestern Medical Center, 5323 Harry Hines Blvd., Dallas, TX 75390 USA

**Keywords:** Systemic lupus erythematous, Whole-genome transcription, DNA methylation, Multiplex cytokine assay, Peripheral blood mononuclear cells, Lupus nephritis

## Abstract

**Background:**

Recent achievement in genetics and epigenetics has led to the exploration of the pathogenesis of systemic lupus erythematosus (SLE). Identification of differentially expressed genes and their regulatory mechanism(s) at whole-genome level will provide a comprehensive understanding of the development of SLE and its devastating complications, lupus nephritis (LN).

**Methods:**

We performed whole-genome transcription and DNA methylation analysis in PBMC of 30 SLE patients, including 15 with LN (SLE LN^+^) and 15 without LN (SLE LN^−^), and 25 normal controls (NC) using HumanHT-12 Beadchips and Illumina Human Methy450 chips. The serum proinflammatory cytokines were quantified using Bio-plex Human Cytokine 27-plex assay. Differentially expressed genes and differentially methylated CpG were analyzed with GenomeStudio, R, and SAM software. The association between DNA methylation and gene expression were tested. Gene interaction pathways of the differentially expressed genes were analyzed by IPA software.

**Results:**

We identified 552 upregulated genes and 550 downregulated genes in PBMC of SLE. Integration of DNA methylation and gene expression profiling showed that 334 upregulated genes were hypomethylated, and 479 downregulated genes were hypermethylated. Pathway analysis on the differential genes in SLE revealed significant enrichment in interferon (IFN) signaling and toll-like receptor (TLR) signaling pathways. Nine IFN- and seven TLR-related genes were identified and displayed step-wise increase in SLE LN^−^ and SLE LN^+^. Hypomethylated CpG sites were detected on these genes. The gene expressions for MX1, GPR84, and E2F2 were increased in SLE LN^+^ as compared to SLE LN^−^ patients. The serum levels of inflammatory cytokines, including IL17A, IP-10, bFGF, TNF-α, IL-6, IL-15, GM-CSF, IL-1RA, IL-5, and IL-12p70, were significantly elevated in SLE compared with NC. The levels of IL-15 and IL1RA correlated with their mRNA expression. The upregulation of IL-15 may be regulated by hypomethylated CpG sites in the promotor region of the gene.

**Conclusions:**

Our study has demonstrated that significant number of differential genes in SLE were involved in IFN, TLR signaling pathways, and inflammatory cytokines. The enrichment of differential genes has been associated with aberrant DNA methylation, which may be relevant to the pathogenesis of SLE. Our observations have laid the groundwork for further diagnostic and mechanistic studies of SLE and LN.

**Electronic supplementary material:**

The online version of this article (doi:10.1186/s13075-016-1050-x) contains supplementary material, which is available to authorized users.

## Background

Systemic lupus erythematosus (SLE) is a potentially severe autoimmune disease characterized by intermittent episodes of increased disease activity that require treatment with immunosuppressive agents [1]. Renal involvement affects more than 60 % of patients with SLE, and is a major contributor to morbidity and mortality [2]. Nephritis remains one of the most devastating complications of lupus, and is an important cause of chronic renal failure despite the availability of new therapeutic regimens [3]. Optimizing the management of lupus nephritis (LN) is therefore important, both to reduce the healthcare burden to society and to improve the outcome of patients [4]. SLE should be closely monitored for renal manifestations, early diagnosis and treatment are essential for renal preservation [5]. The most commonly used marker of renal disease is urinary protein. Spot urinary protein-to-creatinine ratio is currently recommended as a marker. However, it is not sufficiently sensitive to detect early nephritis. Conventional markers of active renal disease are serum levels of anti-dsDNA, anti-C1q antibody, and complement levels. However, the sensitivity of these markers varied greatly depending on the assay system used. The specificity ranges between 50 and 75 % for active renal disease [6, 7]. A better understanding of the pathogenesis of LN is an important step in identifying more biomarkers and targeted therapeutic approaches [8].

Substantial research has helped define the pathogenic mechanisms of SLE renal manifestations [9]. A number of genetic polymorphisms have been associated with lupus, it is now recognized that multiple genes, each making only a small contribution, account for the risk of developing SLE. Several genome-wide scans have allowed major advances in the identification of genetic regions predisposing to SLE, bringing the total number of validated loci to more than 50 [10]. However, many nongenetic factors (such as environment) have now been linked to lupus, environmentally induced epigenetics may also play a critical role in autoimmune immunopathology [11]. Global epigenetic modification, such as DNA methylation and chromatin modification, are directly influenced by the environment, and play an important role in the pathogenesis of SLE [12]. Epigenetic alterations have an important effect on gene expression regulation. Many studies have investigated the association between genetics, DNA methylation, and gene expression in SLE [13–16]. In previous work, hypomethylation of global genomic DNA has been demonstrated in several autoimmune-related gene promoters in isolated T cells from SLE. Genes with aberrant DNA demethylation identified in lupus T cells include ITGAL, CD70, PRF1, and the X chromosome gene CD40LG [17–21]. Similar data are not yet available for LN, and most of the association studies to date have yielded inconsistent results across populations [22].

In the present study, we analyzed the DNA methylation distribution and the relevant mRNA expression variation in peripheral blood mononuclear cells (PBMC) from SLE patients with or without LN using whole-genome DNA methylation and gene expression arrays. We also profiled the levels of inflammatory cytokines in sera of SLE patients by bead-based multiplex assays. The integration of genome-wide gene transcription and DNA methylation expended our insight into the mechanisms affecting epigenetic alteration, gene expression, and LN susceptibility. Combined with the cytokine expression, our data provide a unique resource for identifying biomarkers that may be useful in the diagnosis of lupus nephritis.

## Methods

### SLE patients and controls

We studied 30 SLE patients (15 SLE with lupus nephritis (LN^+^) and 15 SLE without lupus nephritis (LN^−^)) and 25 normal controls (NC). Patients and controls were matched for age, sex, and ethnicity. All SLE patients meet the American College of Rheumatology (ACR) classification criteria for SLE [23]. The clinical features of SLE patients included in this study are shown in Table 1. SLE LN^+^ is defined as clinical and laboratory manifestations that meet ACR criteria (persistent proteinuria >0.5 g/day or greater than 3+ by dipstick, and/or urine cellular casts including red blood cells (RBC), hemoglobin, granular, tubular, or mixed) [2, 23]. A review of the ACR criteria has recommended that “active urinary sediment” (>5 RBCs/high-power field (HPF), >5 white blood cells (WBC)/HPF in the absence of infection) can be substituted for cellular casts [24]. The patients enrolled in the study did not have lymphopenia or cytopenia in previous history. This study was approved by the institutional review board at Xiangya Hospital, Central South University of China (Changsha, Hunan, China). All the participants in the study signed a written informed consent prior to participation [2].

**Table 1:** Clinical manifestation and laboratory data of SLE patients and normal controls

Clinical characteristics	NC (*n* = 25)	SLE LN^−^ (*n* = 15)	SLE LN^+^ (*n* = 15)	*p* value^d^		
				SLE LN^−^ vs. NC	SLE LN^+^ vs. NC	SLE LN^+^ vs. SLE LN^−^
Age (mean + SD)	32.2 ± 9.04	28.2 ± 6.7	30.13 ± 7.68	n.s.	n.s.	n.s.
Sex (M/F)	5/20	2/13	2/13	n.s.	n.s.	n.s.
Disease duration (months)	-	4.90 ± 4.18	3.33 ± 4.21	-	-	n.s.
SLEDAI	-	9.06 ± 4.06	14.93 ± 4.65	-	-	0.001
1997 ACR classification criteria for SLE						
Malar rash	-	8	11	-	-	-
Discoid rash	-	1	3	-	-	-
Photosensitivity	-	4	3	-	-	-
Oral ulcers	-	2	1	-	-	-
Nonerosive arthritis	-	8	6	-	-	-
Pleuritis or pericarditis	-	1	3	-	-	-
Renal disorder	-	0	15	-	-	-
Neurological disorder	-	1	0	-	-	-
Hematological disorder	-	2	7	-	-	-
Immunological disorder	-	11	12	-	-	-
Positive antinuclear antibody (ANA)	-	15	14	-	-	-
WBC (×10^9^/L)^a^	-	6.64 ± 0.70	6.45 ± 0.90	-		0.866
Neutrophils (×10^9^/L)^b^	-	5.00 ± 0.69	4.74 ± 0.76	-		0.799
Lymphocytes (×10^9^/L)^c^	-	1.54 ± 0.21	1.22 ± 0.18	-		0.246
C3 (mg/dL)	-	534.5 ± 280.1	293.2 ± 196.2	-		0.0108
C4 (mg/dL)	-	133.2 ± 147.4	88.02 ± 86.48	-		0.3145

There were no significant difference in age and gender between normal controls and SLE. SLE LN^+^ patients exhibited higher disease activity (SLEDAI score) and lower level of complement C3 than SLE LN^-^. Complete blood count (CBC) in SLE patients showed that the count of white blood cells (WBC), neutrophils, and lymphocytes are within the normal range in SLE patients

*SLE* systemic lupus erythematosus, *NC* normal controls, *SLE LN* ^*+*^ SLE with lupus nephritis, *SLE LN* ^*−*^ SLE without lupus nephritis, *SLEDAI* SLE Disease Activity Index, *n.s.* not significant, *ACR* American College of Rheumatology, *C3* complement component 3, *C4* complement component 4

^a^Normal range of white blood cell (WBC) count: 3.5–10.5 × 10^9^/L

^b^Normal range of neutrophils: 2.0–7.0 × 10^9^/L

^c^Normal range of lymphocytes: 1.0–3.0 × 10^9^/L

^d^ *t* test, *p* < 0.05 means significant

### DNA and RNA isolation

Peripheral blood samples were obtained from SLE patients and normal controls. The PBMC were isolated from heparinized blood by density gradient centrifugation over Ficoll-Paque Plus (GE Healthcare, Piscataway, NJ, USA). Total RNA was isolated from PBMC by standard phenol–chloroform extraction using Trizol reagent (Invitrogen Life Technologies, Carlsbad, CA, USA) according to the manufacturer’s instructions and the concentration was measured on Nanodrop ND-1000 Spectrophotometers (Thermo Fisher Scientific, Waltham, MA, USA). RNA quality was checked on Bioanalyzer Nanochip (Agilent Technologies, Santa Clara, CA, USA) and the samples with RNA integrity number (RIN) >7 were required for microarray analysis. Genomic DNA was isolated from whole blood cells using genomic DNA extraction kits (Life Technologies, Gaithersburg, MD, USA) and DNA integrity was analyzed by agarose gel electrophoresis.

### Genome-wide DNA methylation analysis

DNA methylation status of 485,000 CpG sites across the whole genome was analyzed using the Illumina HumanMethylation 450 BeadChip array. The array covers 99 % of RefSeq genes, with an average of 17 CpG sites per gene region distributed across the promoter, 5′UTR, first exon, gene body, and 3′UTR. It covers 96 % of CpG islands, with additional coverage in island shores and the regions flanking them (Illumina, Inc., San Diego, CA, USA). Genomic DNA (1 μg) extracted from PBMC was bisulfite converted using EZ DNA Methylation kit (Zymo Research Corp, Orange, CA, USA. cat. no. D5004), and utilizing a cyclic denaturation step during the conversion reaction. Four microliters of bisulfite-converted DNA were used for hybridization on Infinium HumanMethylation 450 BeadChip kit. According to the Illumina Infinium HD Methylation protocol, the following steps were performed, whole-genome amplification, end-point fragmentation, precipitation, and resuspension. The resuspended samples were hybridized onto HumanMethylation 450 BeadChips at 48 °C for 18 hours. Then unhybridized and nonspecifically hybridized DNA were washed away, followed by a single nucleotide extension and repeated rounds of staining. Finally, the BeadChip was washed, coated, and scanned. After scanning, the intensities of images were extracted using GenomeStudio Methylation module software (Illumina, Inc.). The methylation score for each CpG was represented as a β value according to the fluorescent intensity ratio. A β value may take any value between 0 (nonmethylated) and 1 (completely methylated). Expression values were extracted, filtered, and normalized using an R package (RnBeads) [25]. The normalized methylation data were analyzed by significance analysis of microarrays (SAM, Stanford University) [26]. The significant differentially methylated sites with FDR <0.05, fold change >1.2 were selected.

### Gene expression studies

Illumina HumanHT-12 v4.0 Expression Beadchips (Illumina, Inc.) were used for mRNA transcription profiling. The platform contains 47,323 transcripts. Each RNA sample was amplified using the Illumina TotalPrep RNA Amplification kit (Thermal Fisher Scientific) with biotin-UTP (Enzo Life Sciences, Inc., Farmingdale, NY, USA) labeling. The TotalPrep Illumina RNA Amplification kit uses T7-oligo(dT) primer to generate single-stranded cDNA followed by a second-strand synthesis to generate double-stranded cDNA, purifies through spin column, uses T7 RNA polymerase to synthesize biotin-labeled cRNA, and purifies again. The cRNA was then quantified using the ND-1000 Spectrophotometer (Thermo Fisher Scientific). A total of 1.5 μg cRNA from each sample was hybridized on each array using standard Illumina protocols with streptavidin-Cy3 (Amersham Biosciences Corp., Piscataway, NJ, USA) being used for detection. Slides were scanned on an Illumina HiScan scanner and analyzed using GenomeStudio (Illumina, Inc.). The raw files of Illumina HumanHT-12 v4.0 Expression array were extracted and normalized (Quantile) in GenomeStudio Module. Normalized raw data were filtered by detection *p* value and median centered by using the Genespring software (Silicon Genetics, Redwood City, CA, USA). Expression data was performed by *t* test without any error correction on the samples. Genes were considered statistically significant at *p* < 0.05.

### Multiplex cytokine assays

Diluted plasma specimens were prepared for analysis in a 96-well plate utilizing the Bio-plex Human Cytokine 27-plex assay (Bio-Rad Laboratories, Veenendaal, The Netherlands) according to the manufacturer’s instructions. The 27-plex assay kit contains beads conjugated with monoclonal antibodies specific for interleukin (IL)-17A, regulated on activation, normal T cell expressed and secreted (RANTES), platelet-derived growth factor-BB (PDGF-BB), IL-2, IL-4, IL-13, basic fibroblast growth factor (bFGF), macrophage inflammatory protein 1 alpha (MIP-1α), IFN-gamma-inducible protein 10 (IP-10), monocyte chemoattractant protein 1 (MCP-1), granulocyte colony-stimulating factor (G-CSF), Eotaxin, tumor necrosis factor alpha (TNF-α), IL-6, IL-9, IL-15, granulocyte-macrophage colony-stimulating factor (GM-CSF), IL-1RA, IL-5, IL-12p70, interferon (IFN)-ϒ, IL-1β, IL-7, IL-8, MIP-1β and vascular endothelial growth factor (VEGF). Analyses were quantified using a Magpix analytical test instrument, which utilizes xMAP technology (Luminex Corp., Austin, TX, USA) and xPONENT 4.2 software (Luminex Corp.). Concentrations of cytokines (pg/ml) were determined on the basis of the fit of a standard curve for mean fluorescence intensity versus pg/ml.

### Real-time PCR validation of the differential gene expression

Differentially expressed genes were validated using Taqman assays on a 7900HT Fast Real-Time PCR system (Applied Biosystems, Foster City, CA, USA). The assay was performed by using a TaqMan RNA-to-CT 1-Step kit (Applied Biosystems) in a total volume of 20 μl, which contained a final concentration of 900 nM sense and antisense primers, 250 nM Taqman gene probe, 1 × TaqMan RT Enzyme Mix, and 1 × TaqMan RT-PCR Mix. The cDNA amplification were monitored using 7900HT Fast Real-Time PCR system under the conditions of 48 °C for 15 min, 95 °C for 10 min, and 40 cycles of 95 °C for 15 s and 60 °C for 1 min. This assay was carried out in triplicate for each sample, including a no-template control. The relative quantity (RQ) of the gene expression in each sample was calculated by normalizing to housekeeping gene GAPDH.

### Pathway analysis

The Ingenuity Pathway Analysis (IPA) tool was used to identify gene networks, functions, and canonical pathways. The IPA software was used for its ability to analyze mRNA data in the context of known biological response and regulatory networks. The score for each network was derived from a *p* value that indicates the expected likelihood of the focus genes being present in a network compared with that expected by chance. The software determines the probability that each biological function assigned to that data set was due to chance alone (http://www.ingenuity.com/).

### Statistical analysis

Data are shown as mean values and standard errors of the means. The univariate comparisons were done using a one-way ANOVA or two-sample *t* test, and multivariable adjustments were done using ANCOVAR to simultaneously adjust for age, SLE Disease Activity Index (SLEDAI) score, and the health status satisfaction score. All *p* values were from two-sided tests. Correlations between pairs of continuous variables were done using Pearson’s R. Dichotomized variables were compared using Fisher’s exact chi-square test. Bonferroni correction was applied in some of the analyses. *p* values of < 0.05 were considered significant. All analyses were performed using SAS version 9.3 software (SAS Institute Inc., Cary, NC, USA). Graphics were carried out using Prism version 6.0 (GraphPad, San Diego, CA, USA).

## Results

### Aberrant mRNA transcription in SLE

**Fig. 1:**
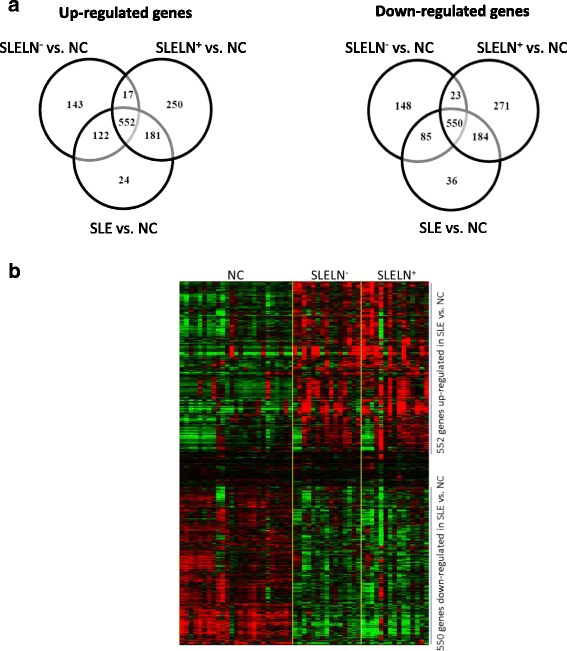
Differentially expressed genes in the PBMC of SLE patients compared with normal controls. **a** Venn diagram shows the number of upregulated genes (*left panel*) and downregulated genes (*right panel*) in three comparisons: SLE vs. NC, SLE LN^−^ vs. NC, and SLE LN^+^ vs. NC. There are 552 upregulated and 550 downregulated genes in common in all three comparisons. **b** Heat map shows hierarchical clustering of the commonly up- and downregulated genes in the three comparisons. *NC* normal controls, *SLE* systemic lupus erythematosus, *SLE LN* ^+^ SLE with lupus nephritis, *SLE LN* ^−^ SLE without lupus nephritis

First, we investigated the gene expression profiles in PBMC of 30 SLE patients (SLE), including 15 SLE with LN (SLE LN^+^) and 15 SLE without LN (SLE LN^−^), and 25 matched normal controls (NC) using Illumina Human HT-12 v4.0 Beadchips. By comparing the gene transcription levels between SLE (including SLE LN^+^ and SLE LN^−^) and NC, we identified 879 upregulated and 855 downregulated genes which are significant (*p* < 0.05) in SLE as compared with NC (Fig. 1a). Further analysis was performed to compare the gene expression differences between SLE LN^+^ vs. NC and SLE LN^−^ vs. NC. There are 1000 upregulated and 1028 downregulated in SLE LN^+^ vs. NC, and 834 upregulated and 806 downregulated genes in SLE LN^−^ vs. NC (Fig. 1a). By Venn diagram analysis, we were able to identify 552 upregulated genes and 550 downregulated genes which are common in all three comparisons (Fig. 1a, Additional file 1: Table S1, and Additional file 2: Table S2), indicating that these common genes are consistently differentially regulated in SLE as compared to the NC group. The profile of the differentially expressed genes in all samples was shown as a heat map in Fig. 1b. The gene expression microarray data has been submitted to the GEO database with accession number GSE81622. The list of the differentially expression genes in each comparison was shown in Additional file 1: Table S1 and Additional file 2: Table S2.

### Aberrant DNA methylation in SLE

**Fig. 2:**
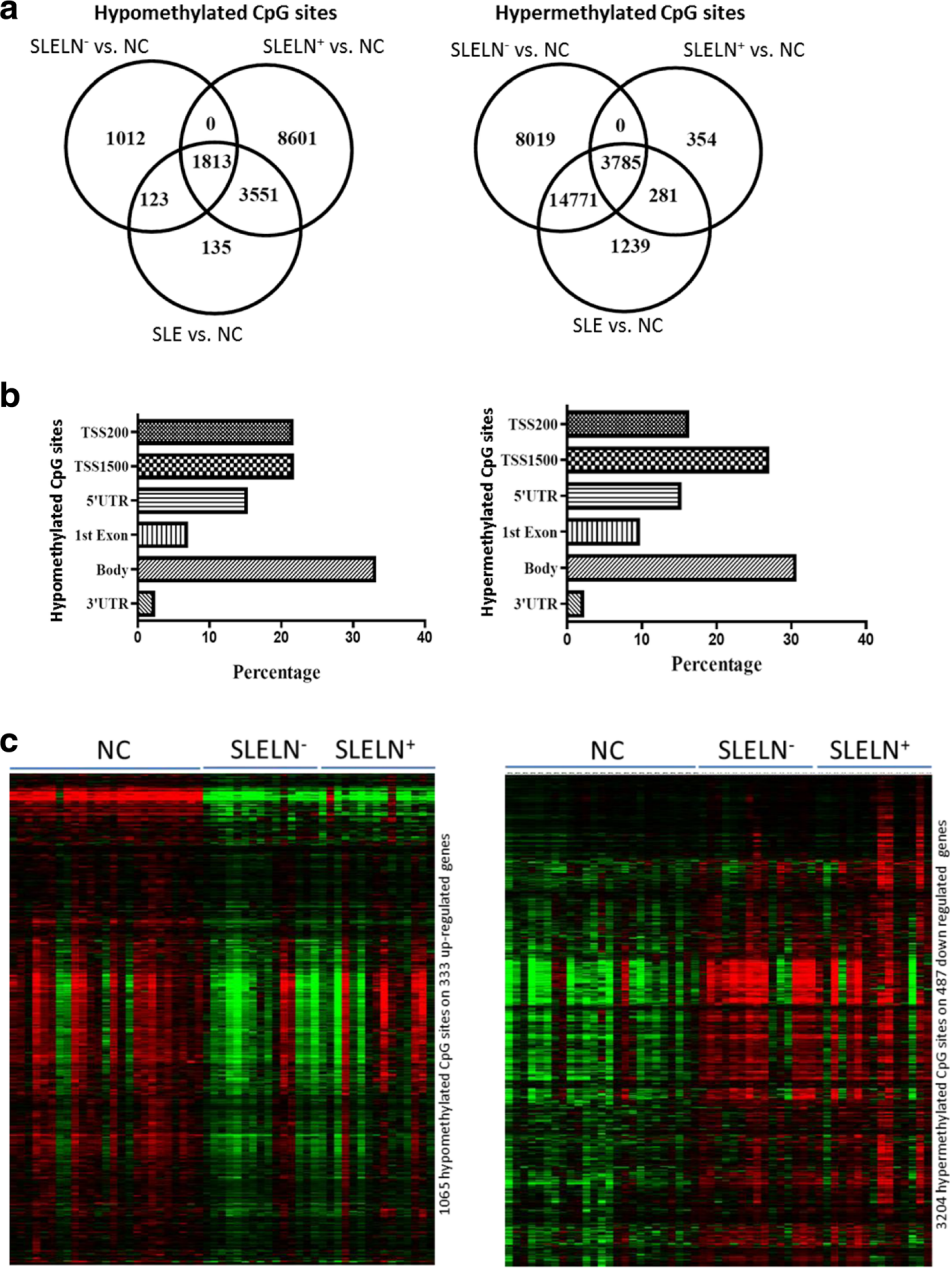
Differentially methylated CpG sites in PBMC of SLE patients compared with normal controls by DNA methylation analysis. **a** Venn diagram showing the number of hypomethylated (*left panel*) and hypermethylated CpG sites (*right panel*) in three comparisons, SLE vs. NC, SLE LN^+^ vs. NC, and SLE LN^−^ vs. NC. A total of 1813 hypomethylated CpG sites and 3785 hypermethylated CpG sites are in common in all three comparisons. **b** The distribution of differentially methylated CpG sites on the genome. The bar charts showed the genomic distribution of hypomethylated (*left panel*) and hypermethylated (*right panel*) probes across different genomic regions: TSS1500 and TSS200 (probes located within 1500 and 200 bp from transcription start site, respectively); 5′UTR region; first exon; gene body and 3′UTR region. **c** Heat maps depicting cluster of the 1065 hypomethylation sites in 333 of 552 upregulated genes, which have one or more hypomethylated CpG sites, and 3024 hypermethylation sites in 487 of 550 downregulated genes, which have one or more hypermethylated sites. *NC* normal controls, *SLE* systemic lupus erythematosus, *SLE LN* ^+^ SLE with lupus nephritis, *SLE LN* ^−^ SLE without lupus nephritis

Next, we performed a global DNA methylation analysis on the same sets of SLE patients and normal controls using a gene chip containing 485,000 CpG sites across the whole genome. Figure 2 showed the numbers of differentially methylated CpG sites in three comparisons: SLE vs. NC, SLE LN^+^ vs. NC, and SLE LN^−^ vs. NC (Fig. 2a). There are 1813 hypermethylated sites and 3785 hypomethylated sites which are common in all three comparisons (Fig. 2a, Additional file 3: Table S3 and Additional file 4: Table S4). The distribution and location of the hypo- and hypermethylated sites are shown is Fig. 2b. Nearly half of the differentially methylated sites (42.95 % of the hypomethylated and 42.99 % for hypermethylated sites) are located on or in the vicinity of the promoter regions, including TSS200 and TSS1500, which is highly suggestive of methylation as a regulatory mechanism on gene transcription. A high percentage of altered methylation sites are also clustered within the gene body, 5′UTR, and first exon (Fig. 2b). The list of the differentially methylated CpG sites in each comparison was shown in Additional file 3: Table S3 and Additional file 4: Table S4. The DNA methylation for all samples has been submitted to the GEO database with accession number GSE81622.

### Correlation of gene expression and DNA methylation in SLE

In order to determine the potential effects of the aberrant DNA methylation on the abnormal gene expression in SLE, we investigated the methylation status of the 552 commonly upregulated genes and 550 commonly downregulated genes in SLE. Among the 552 upregulated genes, 333 genes (60.5 %) have one or more hypomethylated CpG sites (total 1065 CpG site in 333 genes as shown in Fig. 2c, *left panel*; and Additional file 5: Table S5); while among the 550 downregulated genes, hypermethylated CpG sites were found in 479 genes (87.1 %) (Fig. 2c, *right panel*; and Additional file 5: Table S5). Our data suggested that DNA methylation may play an important role in the regulation of gene expression in the development of SLE.

### Functional analysis on the genes differentially expressed in SLE

**Fig. 3:**
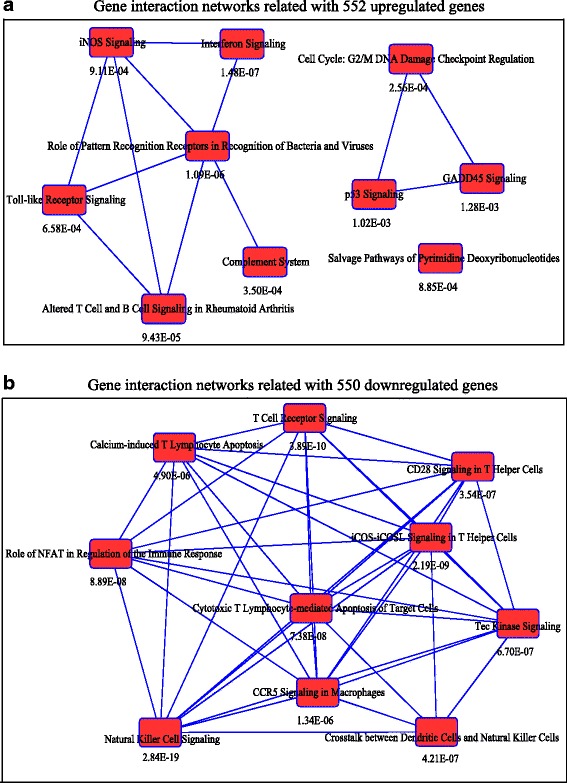
Gene interaction networks among the common up- and downregulated genes in the PBMC of SLE using IPA software. **a** Top ten pathways showing inter-relationships among 552 upregulated genes in SLE. **b** Top ten pathways showing inter-relationships among the 550 downregulated genes in SLE. The top ten pathways are represented by the *red bar*. The lines connecting the bars depict possible gene interactions in the network. The expected likelihood of the gene involved in the pathway was indicated by *p* value in the network

To decipher the molecular pathways associated with dysregulated genes in SLE, we performed Ingenuity Pathway Analysis (IPA) on the differentially expressed genes which are common in all three comparisons. The molecular pathways most significantly correlated with the 552 upregulated genes are showed in Fig. 3a. The top five pathways enriched in the upregulated genes including interferon signaling (1.48E-07), role of pattern recognition receptors in recognition of bacteria and viruses (1.09E-06), altered T cell and B cell signaling in rheumatology arthritis (9.43E-05), cell cycle: G2/M DNA damage checkpoint regulation (2.56E-04) and toll-like receptor signaling (6.58E-04) (Fig. 3a). Figure 3b exhibited the molecular pathways related with the 550 downregulated genes, including natural killer cell signaling (2.84E-19), T cell receptor signaling (3.89E-10), iCOS-iCOSL signaling in T helper cells (2.19E-09), cytotoxic T lymphocyte-mediated apoptosis of target cells (7.38E-08) and role of NFAT in regulation of the immune response (8.89E-08) (Fig. 3b).

### DNA hypomethylation related with the overexpression of IFN-related genes in SLE

**Fig. 4:**
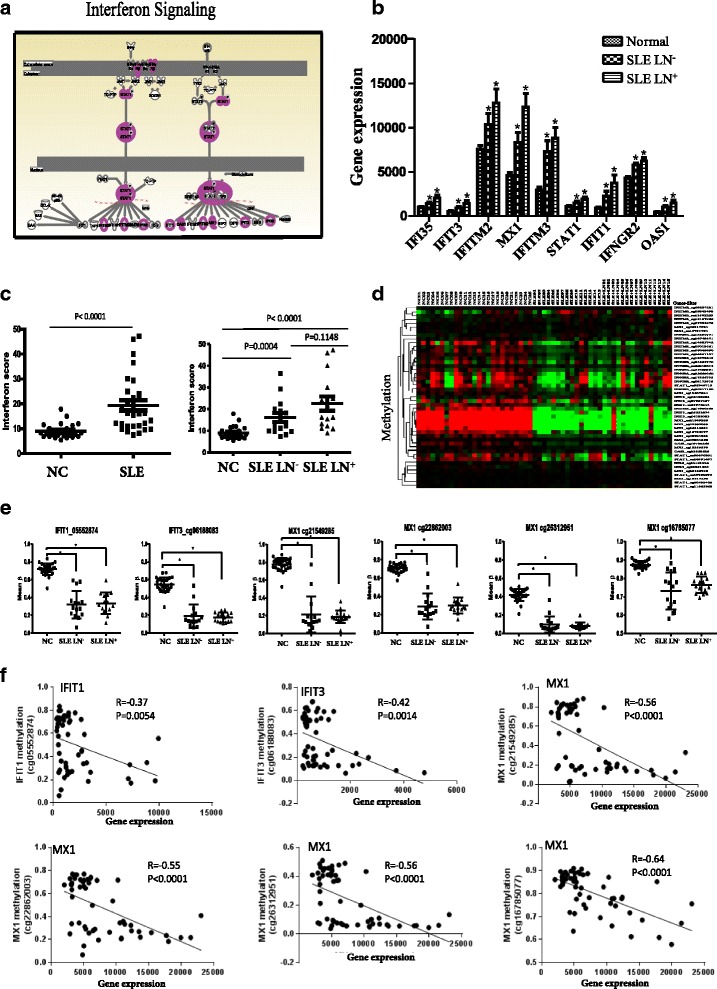
Upregulated genes in PBMC of SLE were significantly enriched in the interferon signaling pathway. **a** A diagram showing the interferon signaling pathway and upregulated genes involved in the network. **b** Array-based mRNA expression represented by normalized intensity in SLE LN^+^, SLE LN^−^, and normal controls.^*^Indicates *p* < 0.05 as compared to normal controls. **c** Interferon scores for SLE patients and controls. The IFN score for each sample was derived from the combined fold change of relative intensity for each gene. The mean intensity in normal controls for each gene was calculated and used as baseline intensity for the gene. Then the intensity of mRNA expression for each gene in patients or controls was normalized with the baseline intensity and resulted in the fold change of relative intensity. The interferon score was a sum of relative intensity for the nine genes (shown in Fig. 5b) that were significantly enriched in the interferon pathway. **d** Hierarchical clustering of the differentially methylated CpG sites on the upregulated genes associated with the interferon signaling pathway. Hypomethylated CpG sites were identified in all the upregulated IFN genes. **e** The CpG sites in MX1, IFIT1 and IFIT3 genes were hypomethylated in SLE patients comparing with NC. ^*^Indicates *p* < 0.05. **f** The mRNA expression of IFN genes (IFT1, IFT3 and MX1) were reversely correlated with the methylation status of the CpG sites in PBMC. *NC* normal controls, *SLE* systemic lupus erythematosus, *SLE LN* ^+^ SLE with lupus nephritis, *SLE LN* ^−^ SLE without lupus nephritis

Among the molecular pathways modulated by aberrantly expressed genes in SLE, type I IFN (IFN-I) signaling pathway showed highest association with the upregulated genes in SLE. The IFN-related genes which are highly expressed in SLE were significantly enriched in this pathway (Fig. 4a). Nine of the IFN-I-related genes, including IFI35, IFIT1, IFIT3, IFITM2, IFITM3, IFNGR2, myxovirus (influenza) resistance 1 (MX1), OAS1 and STAT1, showed a stepwise increase from NC to SLE LN^−^ to SLE LN^+^, although the difference between SLE LN^−^ and SLE LN^+^ on these genes (except MX1) was not statistically significant (Fig. 4b), suggesting that the dysregulation of IFN pathway genes may play an important role in the pathogenesis of SLE. The gene expression data on some IFN genes were further confirmed by real-time PCR and data was shown in Additional file 6: Figure S1. Based on the average expression of these nine IFN genes, we calculated an IFN score for each sample. The mean IFN score for SLE patients (15.23 ± 5.95) was significantly greater than NC (9.00 ± 2.94) (*p* < 0.0001). The IFN score for SLE LN^+^ (22.57 ± 12.84) was slightly higher than SLE LN^−^ (16.13 ± 8.36) but not statistically significant (*p* > 0.1) (Fig. 4c).

In order to understand if the increased expression of IFN signaling genes in SLE were regulated by altered DNA methylation, we examined the methylation status of the CpG sites on the upregulated IFN genes in all NC and SLE samples. Among the 47 CpG sites in the nine IFN-related genes, 41 CpG sites exhibited decreased methylation (hypomethylation) in SLE patients compared with NC on mean β value (*p* < 0.05), including two sites on four genes (IFI35, IFITM2, IFIT1 and OAS1), three on IFIT3, five on IFITM3, six sites on STAT1, eight sites on IFNGR2 and 11 sites on MX1 (Fig. 4d). Shown in Fig. 4e are the six CpG sites which exhibited the greatest methylation change on the IFN genes between SLE and NC, four on MX1 (cg22862203, cg26312951, cg16785077, cg21549285), one on IFIT3 (cg06188083) and one on IFIT1 (cg05552874) (Fig. 4e). The methylation of these sites reversely correlated with the transcription levels of the corresponding genes in PBMC (Fig. 4e, f).

### DNA hypomethylation associated with higher transcription of the genes in the toll-like receptor signaling pathway in SLE

**Fig. 5:**
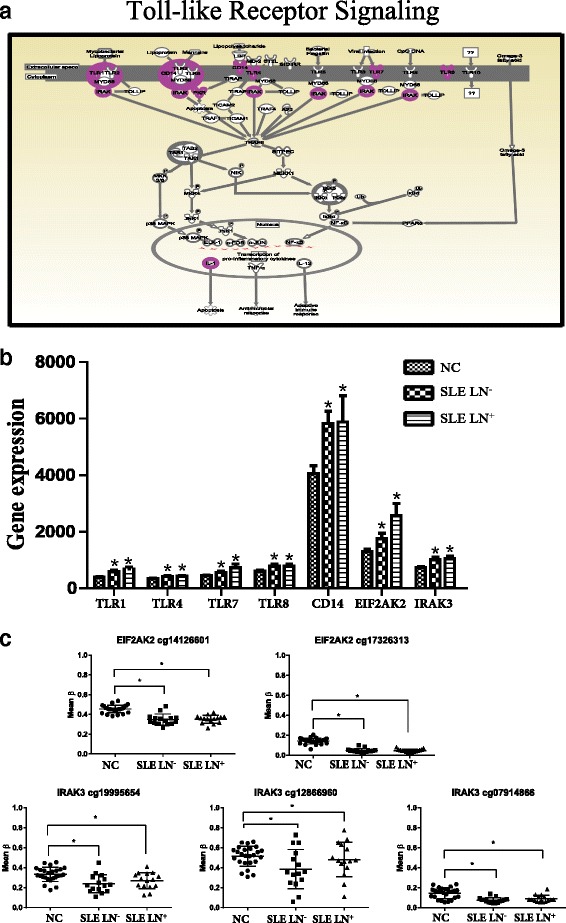
Upregulated genes in PBMC of SLE were significantly enriched in the toll-like receptor signaling pathway. **a** A diagram showing the toll-like receptor signaling pathway and upregulated genes involved in the network. **b** Array-based mRNA expression represented by normalized intensity in SLE LN^+^, SLE LN^−^, and normal controls.^*^Indicates *p* < 0.05 as compared to normal controls. **c** The CpG sites in EIF2AK2 and IRAK3 genes were hypomethylated in SLE patients compared with NC. ^*^Indicates *p* < 0.05. *NC* normal controls, *SLE* systemic lupus erythematosus, *SLE LN* ^+^ SLE with lupus nephritis, *SLE LN* ^−^ SLE without lupus nephritis

In addition to interferon signaling pathway, we noted that the expression of several genes related to the toll-like receptor signaling (TLR) pathway were also elevated in SLE. TLRs play an essential role in the innate immune system and regulate a pro-/anti-inflammatory balance (Fig. 5a). We found that mRNAs for cluster of differentiation 14 (CD14), eukaryotic translation initiation factor 2-alpha kinase 2 (EIF2AK2), IL1RN, interleukin-1 receptor-associated kinase 3 (IRAK3), TLR1, TLR4, TLR7 and TLR8 were significantly upregulated in SLE compared with NC (Fig. 5b). The expression of the TLR7 and EIF2AK2 genes were higher in SLE LN^+^ as compared to SLE LN^−^, but the change was not statistically significant (Fig. 5b).

We then examined the DNA methylation status on the CpG sites of these differentially expressed genes. There are multiple CpG sites (eight on IRAK3, two on EIF2AK2, four on TLR4, three on IL-1RA, two on TLR8 and one on TLR7), which showed different methylation between SLE and NC. The most significantly hypomethylated sites were found on EIF2AK2 (cg14126601 and cg17326313) and IRAK3 (cg19995654, cg12866960, and cg07914866) (Fig. 5c). The analysis showed that the increase in gene expression was inversely correlated with the reduction in DNA methylation level (decreased ∆β) on the same genes, suggesting that DNA hypomethylation may be involved in regulation of gene expression for the TLR pathway in SLE.

### Aberrant mRNA expression in SLE with LN

**Fig. 6:**
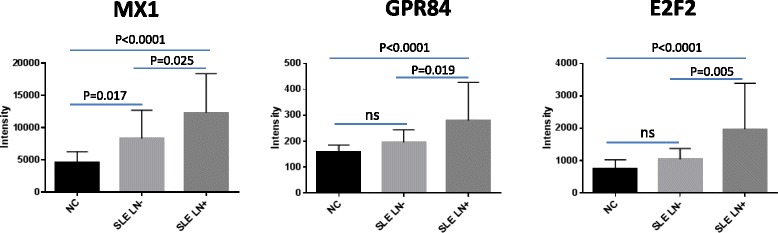
Upregulated genes in PBMC of SLE LN^+^ patients. Three genes were identified to be significantly upregulated in SLE LN^+^ compared with SLE LN^−^ and NC (*p* < 0.05). Expression value represented by normalized intensity in SLE LN^+^, SLE LN^−^, and NC.^*^Indicates *p* < 0.05. *NC* normal controls, *SLE* systemic lupus erythematosus, *SLE LN* ^+^ SLE with lupus nephritis, *SLE LN* ^−^ SLE without lupus nephritis

In order to identify the genes which may play a role in the development of LN, we compared the gene expression profiles in PBMC between SLE LN^+^ and SLE LN^−^ patients. From the 552 common upregulated and 550 common downregulated genes in SLE, we only identified three genes (MX1, G protein-coupled receptor 84 (GPR84), E2F transcription factor 2 (E2F2)), which were significantly increased in SLE LN^+^ compared with both NC and SLE LN^−^ (*p* < 0.05, Fig. 6). We then checked the methylation status of the three upregulated genes and no significant methylation change was identified on these genes between SLE LN^+^ and SLE LN^−^ patients.

### Aberrant level of serum cytokine/chemokine in SLE and their transcriptional regulation

**Fig. 7:**
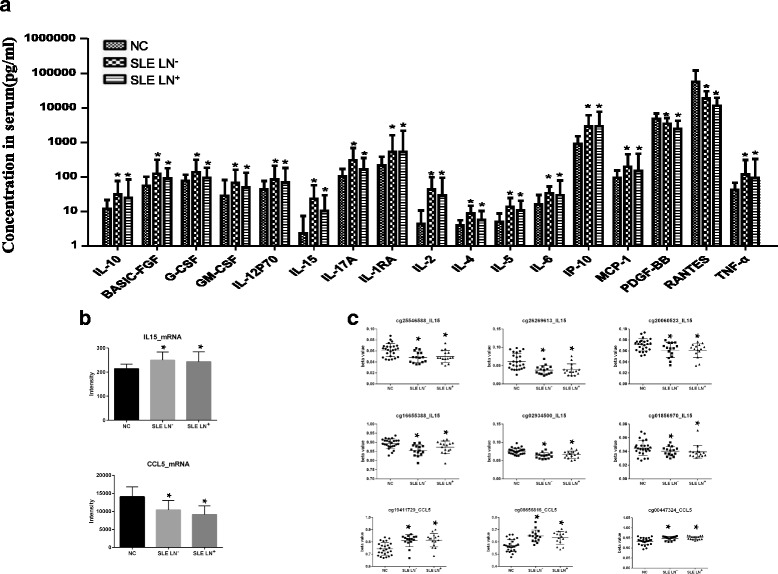
Quantification of 27 cytokines and chemokines in sera of SLE patients and controls using bead-based multiplex assays. **a** Seventeen cytokines showed significant difference in SLE patients compared with NC, 15 were increased and two were decreased in both SLE LN^−^ and SLE LN^+^ compared with NC (^*^ *p* < 0.05). **b** mRNA expression level in PBMC for the cytokines which showed significant difference in sera of SLE patients. IL-15 mRNA was significantly upregulated and CCL5 mRNA were downregulated in PBMC of SLE patients compared with NC (^*^ *p* < 0.05). **c** Six CpG sites in the IL-15 gene were hypomethylated and three CpG sites in CCL5 (RANTES) gene were hypomethylated in PBMC of SLE compared with NC. (^*^ *p* < 0.05). *NC* normal controls, *SLE* systemic lupus erythematosus, *SLE LN* ^+^ SLE with lupus nephritis, *SLE LN* ^−^ SLE without lupus nephritis

Cytokines are immunoregulatory mediators which play important roles in the pathogenesis of SLE. In an effort to understand the transcriptional regulation on serum cytokines, we performed quantitative analysis on 27 cytokines/chemokines in the sera of SLE and NC using a bead-based multiplex assay. Fifteen of the 27 serum factors, including IL-2, IL-4, IL-5, IL-6, IL-10, IL-12p70, IL-15, IL-17A, bFGF, MCP-1, IP-10, G-CSF, GM-CSF, IL-1RA and TNF-α were significantly higher in SLE LN^−^ and SLE LN^+^, compared with NC (Fig. 7a, Additional file 7: Table S6). However, two cytokines, PDGF-BB and RANTES, were significantly lower in the two SLE groups compared with NC (Fig. 7a). There was no significant difference on the serum levels of the cytokines between SLE LN^+^ patients and their SLE LN^−^ counterparts (Fig. 7a, Additional file 7: Table S6). At the transcription level, we found that IL-15 mRNA was elevated in SLE, and RANTES (chemokine (C-C motif) ligand 5 (CCL5)) mRNA was decreased in SLE, compared with NC (Fig. 7b), indicating that the serum level of these two cytokines corresponded with their mRNA expression in PBMC. We further checked the DNA methylation status on IL-15 and CCL5, and identified six of 11 methylation sites (cg01856970, cg02934500, cg25546588, cg16655388, cg26269613, and cg20060523) on IL-15, which are located at 5′UTR, TSS1500, and TSS 200 regions, were hypomethylated in both SLE LN^+^ and SLE LN^−^ patients in relation to NC (Fig. 7c). We also found three CpG sites (cg00447324, cg08656816, and cg19411729) at the 5′UTR region of CCL5 (RANTES) gene were hypermethylated in SLE compared with controls (Fig. 7c).

## Discussion

Multiple studies on a variety of cell types have demonstrated altered gene expression and DNA methylation patterns in autoimmune diseases, providing important new directions toward understanding the pathogenesis of immune-mediated diseases [27, 28]. A number of studies have compared gene expression in PBMC from patients with SLE versus healthy individuals or other autoimmune conditions, which provide substantial advantages in the search for diagnostic and prognostic biomarkers in autoimmune disease [29, 30]. But LN remains a major challenge and one of the most severe manifestations of SLE. Early diagnosis and intervention are essential for favorable patient outcomes [31]. It would be of great help to identify biomarkers so that we could assess LN activity or kidney damage, and predict an imminent exacerbation of renal disease accurately [2].

In this study, we performed an unbiased genome-wide gene transcription and DNA methylation study in PBMC from SLE patients with LN and without LN and compared with matched normal controls. By cross comparison of different SLE groups with NC, we identified a group of genes (552 up- and 550 downregulated) which are consistently differentially expressed in SLE as compared with controls. DNA methylation analysis on the whole-genome level distinguished a large number of CpG sites which were differentially methylated (1813 hypermethylated and 3785 hypomethylated sites) in SLE compared to NC. With integrated analysis of the gene transcription and DNA methylation data, we found that 60.5 % of the upregulated genes contain hypomethylated CpG sites and 87.1 % downregulated genes harbor hypermethylated CpG sites in their regulatory regions of the genome, suggesting that DNA methylation may have regulatory effect on most of the differentially expressed genes in SLE. Profiling mRNA expression and DNA methylation in the same set of SLE and NC samples enable us to investigate the complexity of gene dysregulation in the context of this disease.

Further analysis was performed to elucidate the potential molecular pathways modulated by the aberrantly expressed and methylated genes in SLE. The current analysis was mainly focused on the common upregulated genes with DNA hypomethylation. To our expectation, the upregulated genes in SLE were significantly enriched in immune regulatory pathways such as IFN signaling pathway, TLR signaling pathway, and proinflammatory signaling pathways, which have been reported to be related with pathogenesis in SLE and other autoimmune disorders [32–34]. It is well known that the overexpression of type I IFN-related genes in peripheral blood immune cells is a major SLE signature [35]. However, the pathogenic role of IFNs and their exact regulation in the development of SLE is still under debate. By exploring the globe gene transcription and DNA methylation in PBMC of SLE with different clinical manifestations (with or without LN), our study provided further evidence that the overexpression of a cluster of IFN genes was involved in the activation of the IFN signaling pathway. In addition, we noticed a stepwise increased expression of nine IFN genes from NC to SLE LN^−^ to SLE LN^+^, suggesting a pathogenic role of the IFNs in the development of SLE and LN, even though the difference between SLE LN^−^ and SLE LN^+^ was not statistically significant for all IFNs except for MX1. Interestingly, almost all the overexpressed IFN genes in SLE were associated with multiple hypomethylated CpG sites in the regulatory region of the genes, indicating epigenetic modification may play a major role in the regulation of IFN gene expression. Previous studies have reported the hypomethylation of IFN genes in the CD4+ T cells in SLE [13]. Our study detected the hypomethylated CpG sites in association with upregulation of IFN genes in PBMC of SLE, suggesting the hypomethylation may be a general regulating mechanism in all immune cells in PBMC, including T, B, macrophages, and dendritic cells. Further studies need to be performed on different cell types and different stages of the diseases to elucidate the exact molecular mechanisms of IFN gene regulation.

Toll-like receptor pathway is another molecular pathway identified in our study to be enriched with dysregulated genes in SLE. Seven upregulated genes in both SLE LN^−^ and SLE LN^+^, including TLR1, TLR4, TLR7, TLR8, CD14, EIF2AK2 and IRAK3, were involved in the regulation of TLR pathways. TLR regulation plays an essential role in the pathogenesis of SLE [36]. It is believed that abnormal activation of TLR7/TLR8 or TLR9 by self RNA or DNA in antigen-presenting cells may cause breach of immune tolerance and results in cytokine, IFN-I, and autoantibody production in T, B, and myeloid cells [37–39]. TLR4 have also been shown to induce TNF-α production in monocytes of SLE patients [40, 41]. Substantial studies have been focused on the TLR signaling pathway for exploration of therapeutic target using adjuvants or inhibitors [42, 43]. Our data revealed the aberrant DNA methylation of TLR and its signaling pathway in SLE, implying a novel therapeutic strategy by intervention of methylation in SLE.

Serum cytokines/chemokines are important biomarkers reflecting immune cell activation and inflammatory reaction during the development of SLE [44]. In this study, we measured the serum levels of 27 pre-inflammatory cytokines/chemokines in SLE patients with or without lupus nephritis and compared them with healthy controls. Our analysis identified 15 cytokines/chemokines which significantly increased in the sera of both SLE LN^−^ and SLE LN^+^ patients, as compared with NC. The increased levels of Th1 cytokines (IL-2, IL-12, IL-15, TNF-a), and Th2 cytokines (IL-4, IL-5, IL-6, IL-10,) reflecting the activation of both Th1 and Th2 T helper cell subsets, which is in agreement with previous studies showing that IL-12, IL-10, IL-6 and TNF-α were correlated with active SLE [45–48]. We also observed the increased level of IL-17, which is secreted by Th17 cells and involved in T cell activation and autoantibodies in SLE [49–51]. It is not surprising that we could not find significant difference on any of the serum cytokines between SLE LN^+^ and SLE LN^−^ in our sample set. Although several cytokines, including MCP-1, IP-10 and IL-6, have been reported to have increased expression in the kidney and urine of SLE patients with active LN compared with no renal SLE, the serum level were not different between SLE patients with or without LN [52], implying that the serum levels of inflammatory cytokines/chemokines are not reflecting the LN activity in SLE patients.

Our data also showed that RANTES (CCL5) and platelet-derived growth factor-BB (PDGF-BB) were significantly lower in both SLE LN^−^ and SLE LN^+^ compared with NC. RANTES, as a proinflammatory chemokine, has been extensively studied and reported to be increased in SLE patients [53, 54]. The discrepancy of our data with previous studies may be due to sample variation and different method used. PDGF-BB is a platelet-derived factor involved in cell proliferation. Its role in SLE has not been reported before.

The regulation of the serum cytokines/chemokines in SLE on the genome level was not fully understood. By analyzing the mRNA expression and the DNA methylation of the cytokine/chemokine genes, we were able to find out if the increased serum level of cytokines in SLE is associated with their mRNA transcription and DNA methylation in the PBMC of the patients. To our surprise, we only found two out of the 17 differentially expressed cytokines, the IL-15 and RANTES, in which the mRNA expression in PBMC correspond with their serum levels. The discrepancy between serum protein level and mRNA for most of the cytokines/chemokines may be due to the cytokine gene expression regulation is cell-type specific and the differential expression may not be detected in PBMC, which is a mixture of different cell types. On the other hand, the increased mRNA transcription on IL-15 in SLE PBMC suggesting the transcription of this cytokine may be activated in multiple cell types. Indeed, IL-15 can be produced by macrophage/monocytes, activated NK cells, CD4+ T cells, and B cells, and involved in the pathogenesis of SLE and other autoimmune diseases [55–57]. Furthermore, the hypomethylation on several CpG sites in the regulatory region of this gene suggested a strong epigenetic regulation of IL-15 in SLE. In addition, our study showed that the lower level of RANTES in serum is correlated with lower mRNA expression in PBMC in SLE patients. Interestingly, the hypermethylated CpG sites at the 5′UTR region of CCL5 (RANTES) gene in SLE patients also indicated a suppressive effect of epigenetic regulation on this gene. This data is currently under validation in a larger sample cohort.

LN is one of the most devastating complications of SLE, but the molecular basis for this disease is not yet clear. Gene expression profiling on PBMC and different subsets of blood cells have identified candidate genes that might contribute to the pathogenesis of SLE and its complications [7, 58, 59]. However, the role of the differentially expressed genes identified from peripheral blood of SLE patients in the pathogenesis of LN is still under debate. By comparing the gene expression profiles in PBMC from SLE LN^−^ and SLE LN^+^ patients, we identified three genes which are significantly elevated in SLE LN^+^ patients, namely, MX1, GPR84 and E2F2. MX1 is one of the IFN-I-inducible genes which have been extensively studied in SLE. Increased MX1 gene expression has been detected in both PBMC and renal intrinsic cells of lupus nephritis patients suggesting its role in the pathogenesis of LN [60, 61]. Our data consolidated previous finding by detecting the upregulation of MX1 on transcription in PBMC and in serum in SLE LN^+^ patients. Furthermore, by analyzing DNA methylation of this gene, we postulated that the higher expression of MX1 in SLE may be regulated by hypomethylation of the CpGs in the regulatory region.

E2F2 and GPR84 are novel biomarkers which have not been previously reported to be associated with SLE or LN. E2F2 is a member of E2F transcription factor family. As a cell cycle activator, E2F2 can regulate the gene expression that govern the cell cycle and DNA synthesis, and thus cell proliferation [62, 63]. There was a study that showed that E2F2 can promote the expression of microRNA let-7a by binding to its promoter and let-7a has been shown to contribute to hyperplasia and the proinflammatory response in SLE [64]. Therefore, the increased expression of E2F2 may associate with the inflammation in SLE and LN. GPR84 is a G protein-coupled receptor for sensing free fatty acid and active inflammatory reaction. Activation of GPR84 by its ligand could elicit chemotaxis of polymorphonuclear leukocytes (PMNs) and macrophages and amplified lipopolysaccharide (LPS)-stimulated production of the proinflammatory cytokine IL-8 from PMNs and TNF-α from macrophages [65]. Further analysis on the expression of these two genes in different cell types and the molecular pathways is warranted to elucidate their role in the pathogenesis of SLE and LN.

Although our current analysis was primarily focused on the genes which were upregulated and hypomethylated in SLE, the identification of a large amount of downregulated genes which were hypermethylated in the SLE genome by globe gene expression and DNA methylation analysis implying that downregulation or silencing of gene expression in PBMC may play an equally important role in SLE and LN [66–70]. Previous studies have demonstrated that the downregulation of IL-2 [35, 67, 71], IL-17 [66], and Notch-1 [69] in SLE T lymphocytes was due to the binding cAMP-responsive element modulator (CREM)-α to the promoter of the genes followed by enhanced CpG DNA methylation. We therefore examined methylation status on the CpG sites of these genes in our study. By comparing the mean β value between NC and SLE (both with LN and without LN involvement), we found that four out of 70 sites on Notch-1 gene, two out of ten sites on IL-17A gene and one out of two sites on IL-2 gene were hypermethylated in SLE (*p* < 0.05) (data not shown). Further analysis on the SLE downregulation gene data set is going on with the expectation to find out more dysregulated pathways modulated by DNA hypermethylation.

There are some limitations in the current study. First, the gene expression and DNA methylation profiling were only performed in PBMC. Given the highly complex nature of this disease, different cell subsets in peripheral blood may have different transcription and methylation profiles during the development of the diseases. However, previous studies using different cell types have identified specific gene signatures from different cell types. These cell type-specific genes allowed a correct classification of PBMC independent from their heterogenic cellular composition. Therefore, based on the expression of cell type-specific signature genes, we should be able to normalize the expression profile of PBMC from all individuals [72]. On the other hand, the advantage of using PBMC includes easy accessibility and less blood volume requirement. The result from PBMC has reflected a general picture of profiling alteration in whole blood. It is therefore ideal for biomarker screening [73]. The second limitation is that the sample size in this study is relatively small. The statistical power may not be strong enough to draw conclusions on some of the differential genes and pathways. The current data will need to be further validated in a larger sample cohort. Third, functional analysis will need to be conducted to confirm the genetic and epigenetic interactions on the dysregulation of genes and pathways in SLE.

## Conclusions

In conclusion, we integrated the gene expression, DNA methylation, and cytokine profiling data in PBMC of SLE patients with and without lupus nephritis. Our study has demonstrated that significant number of differential genes in SLE were involved in IFN, TLR signaling pathways, and inflammatory cytokines. The enrichment of differential genes has been associated with aberrant DNA methylation, which may be relevant to the pathogenesis of SLE. Our observations have laid the groundwork for further diagnostic and mechanistic studies of SLE and LN.

## Abbreviations

ACR, American College of Rheumatology; bFGF, basic fibroblast growth factor; CCL5, chemokine (C-C motif) ligand 5; CD14, cluster of differentiation 14; E2F2, E2F transcription factor 2; EIF2AK2, eukaryotic translation initiation factor 2-alpha kinase 2; G-CSF, granulocyte colony-stimulating factor; GM-CSF, granulocyte-macrophage colony-stimulating factor; GPR84, G protein-coupled receptor 84; HPF, high-power field; IFN, interferon; IL, interleukin; IP-10, IFN-gamma-inducible protein 10; IPA, Ingenuity Pathway Analysis; IRAK, interleukin-1 receptor-associated kinase; LN, lupus nephritis; MCP-1, monocyte chemoattractant protein; MIP-1, macrophage inflammatory protein 1; MX1, myxovirus (influenza) resistance 1; NC, normal controls; PBMC, peripheral blood mononuclear cell; PDGF, platelet-derived growth factor-BB; RANTES, regulated on activation, normal T cell expressed and secreted; SLE, systemic lupus erythematosus; SLEDAI, SLE Disease Activity Index; SLE LN^+^, SLE with lupus nephritis; SLE LN^−^, SLE without lupus nephritis; TLR, toll-like receptor; TNF-α, tumor necrosis factor alpha

## Additional files

Additional file 1: Table S1. Upregulated genes in PBMC of SLE patients compared with normal controls by microarray analysis. S1-1 showed the complete list of 552 upregulated genes in SLE. Three group comparisons in gene expression, SLE (LN^−^ and LN^+^) vs. NC (S1-2), SLE LN^−^ vs. NC (S1-3), and SLE LN^+^ vs. NC (S1-4), were performed to identify upregulated genes by fold change (FC) > 1.2 and *p* < 0.05 within each group. The 552 common upregulated genes in SLE were then selected from each of the three group comparisons. S1-5 showed gene expression of 552 common upregulated genes represented by normalized intensity in SLE. (XLSX 694 kb) Additional file 2: Table S2. Downregulated genes in PBMC of SLE patients compared with normal controls by microarray analysis. S2-1 showed the complete list of 550 downregulated genes in SLE. Three group comparisons, SLE (LN^−^ and LN^+^) vs. NC (S2-2), SLE LN^−^ vs. NC (S2-3), and SLE LN^+^ vs. NC (S2-4), were performed to identify downregulated genes by fold change (FC) > 1.2 and *p* < 0.05 within each group. The 550 common downregulated genes in SLE were then selected from the genes identified in each of the three group comparisons. S2-5 showed gene expression of 550 common downregulated genes represented by normalized intensity in SLE. (XLSX 710 kb) Additional file 3: Table S3. Hypermethylated CpG sites in PBMC of SLE patients comparing normal controls by genome-wide DNA methylation analysis. Three group comparisons, SLE LN^+^ vs. NC (S3-1), SLE LN^−^ vs. NC (S3-2), and SLE (LN^−^ and LN^+^) vs. NC (S3-3), were performed to identify hypermethylated DNA CpG sites by fold change (FC) > 1.2 and q value < 5% within each group. The 1813 common hypermethylated sites identified in each of the three group comparisons were then selected and listed in S3-4. (XLSX 814 kb) Additional file 4: Table S4. Hypomethylated CpG sites in PBMC of SLE patients comparing normal controls by genome-wide DNA methylation analysis. Three group comparisons, SLE LN^+^ vs. NC (S4-1), SLE LN^−^ vs. NC (S4-2), and SLE (LN^−^ and LN^+^) vs. NC (S4-3), were performed to identify hypomethylated CpG sites by fold change (FC) > 1.2 and q value < 5% within each comparison. The 3785 common hypomethylated sites identified in each of the three group comparisons were then selected and listed in S4-4. (XLSX 1843 kb) Additional file 5: Table S5. Hypomethylated or hypermethylated CpG sites associated with the commonly up- or downregulated genes in SLE. S5-1 showed a complete list of 1065 hypomethylated CpG sites that were associated with 333 upregulated genes in SLE, and the normalized β values of each site for NC and SLE samples. S5-2 showed 3024 hypermethylated CpG sites that were associated with 478 downregulated genes, and the normalized β values of each site for NC and SLE samples. (XLSX 2264 kb) Additional file 6: Figure S1. Validation on the expression of five IFN-related genes in PBMC of a different cohort of SLE patients and controls by real-time PCR. The Ct value for IFI35, IFI44, MX1, STAT1, and IFIT1 was normalized with housekeeping gene GAPDH for each sample. Relative quantity (RQ) represents the relative level of gene expression in SLE and NC. (^*^Indicates significant difference between NC and SLE, *p* < 0.05). (PDF 496 kb) Additional file 7: Table S6. Profile 27 cytokine/chemokine from sera of SLE patients and normal controls. Serum cytokines were quantitatively measured using Bio-Plex Pro™ Human Cytokine 27-plex assay kits. The data represented the mean concentration for each cytokine in the sera of NC, SLE LN^−^, or SLE LN^+^ patients. (PDF 479 kb)
