# Supplementary figures and images for: Whole-genome transcription and DNA methylation analysis of peripheral blood mononuclear cells identified aberrant gene regulation pathways in systemic lupus erythematosus
Source: Arthritis Res Ther. 2016 Jul 13;18:162. doi: 10.1186/s13075-016-1050-x (PMC4942934; doi:10.1186/s13075-016-1050-x)

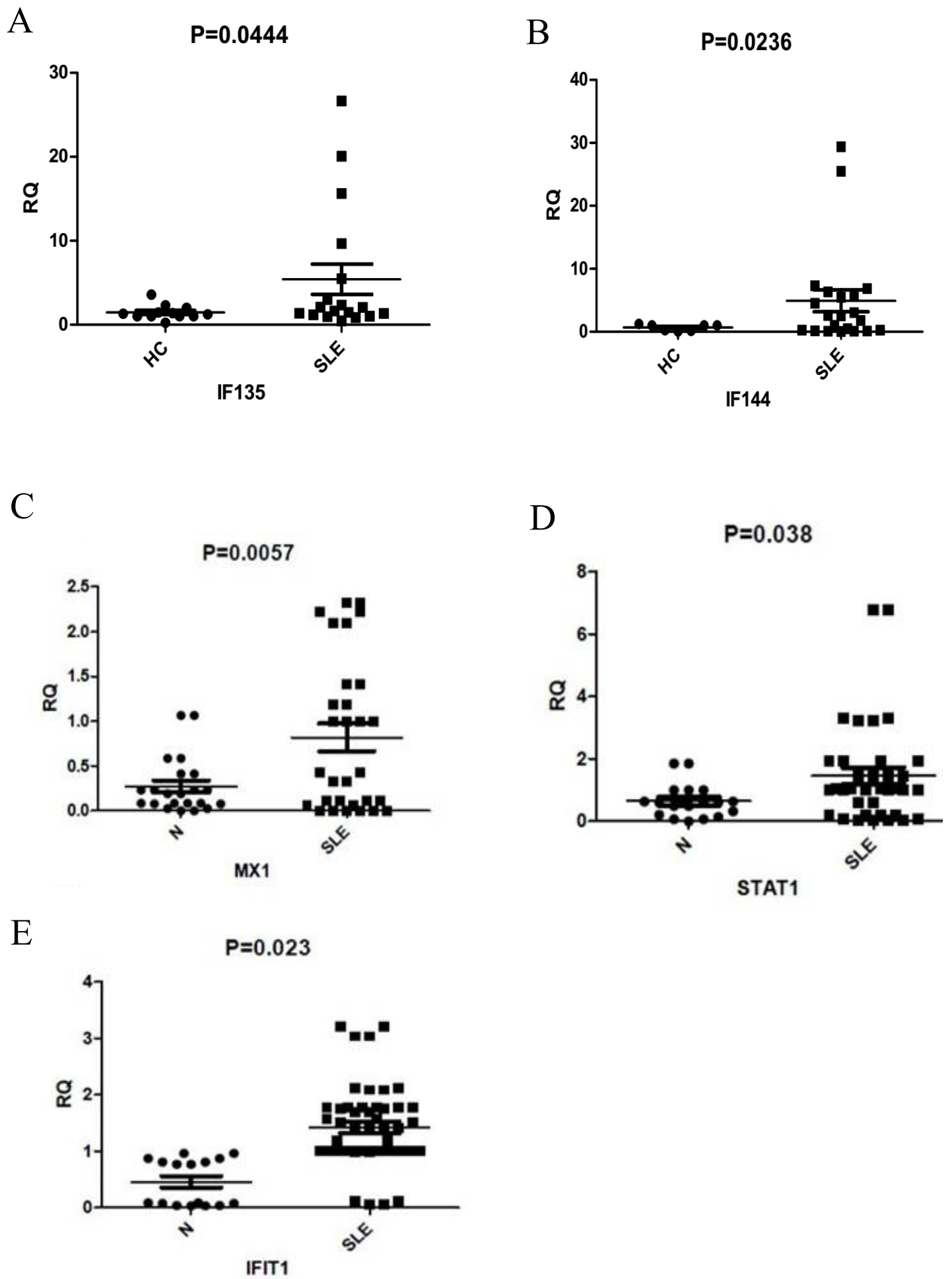

Figure S1

Supplement: Additional file 6: Figure S1. — Validation on the expression of five IFN-related genes in PBMC of a different cohort of SLE patients and controls by real-time PCR. The Ct value for IFI35, IFI44, MX1, STAT1, and IFIT1 was normalized with housekeeping gene GAPDH for each sample. Relative quantity (RQ) represents the relative level of gene expression in SLE and NC. (*Indicates significant difference between NC and SLE, p < 0.05). (PDF 496 kb) [file 13075_2016_1050_MOESM6_ESM.pdf]
