# Supplementary material for: Whole-genome transcription and DNA methylation analysis of peripheral blood mononuclear cells identified aberrant gene regulation pathways in systemic lupus erythematosus
Source: Arthritis Res Ther. 2016 Jul 13;18:162. doi: 10.1186/s13075-016-1050-x (PMC4942934; doi:10.1186/s13075-016-1050-x)
Supplement: Additional file 7: Table S6. — Profile 27 cytokine/chemokine from sera of SLE patients and normal controls. Serum cytokines were quantitatively measured using Bio-Plex Pro™ Human Cytokine 27-plex assay kits. The data represented the mean concentration for each cytokine in the sera of NC, SLE LN−, or SLE LN+ patients. (PDF 479 kb) [file 13075_2016_1050_MOESM7_ESM.pdf]

Table S6. Profiling 27 cytokine/chemokine from sera of SLE patients and normal controls

Cytokines	NC (N=25)[a]	SLE LN$^-$(N=15)[a]	SLE LN$^+$(N=15)[a]	p Value[b]	p Value[c]	p Value[d]
IL-10	11.73±9.71	30.64±44.68	24.19±59.78	0.00	0.01	n.s.
BASIC-FGF	55.41±44.88	123.22±189.72	89.73±88.08	0.00	0.00	n.s.
EOTAXIN	132.43±72.22	161.52±163.69	124.03±123.32	n.s.	n.s.	n.s.
G-CSF	76.79±38.99	136.36±172.98	93.26±88.37	0.00	0.04	n.s.
GM-CSF	28.4±52.19	66.31±93.55	49.26±82.73	0.00	0.02	n.s.
IFN-γ	87.59±80.58	183.89±261.01	140.2±344.05	0.00	n.s.	n.s.
IL-12P70	42.9±32.52	83.59±125.88	68.26±111.45	0.00	0.00	n.s.
IL-13	21.16±32.91	25.23±39.43	20.44±21.79	n.s.	n.s.	n.s.
IL-15	2.29±5.01	23.1±33.54	10.17±19.33	0.00	0.00	n.s.
IL-17A	102.69±65.98	300.36±386.14	162.7±188.17	0.00	0.00	n.s.
IL-1β	4.69±7.39	52.83±303.71	5.82±14.63	0.04	n.s.	n.s.
IL-1RA	213.77±169.56	526.31±1089.11	524.04±1639.91	0.00	0.01	n.s.
IL-2	4.26±6.21	43.2±53.7	28.71±65.82	0.00	0.00	n.s.
IL-4	3.88±1.6	8.63±5.95	5.58±4.69	0.00	0.00	n.s.
IL-5	4.9±3.74	13.68±10.78	10.54±9.88	0.00	0.00	n.s.
IL-6	15.72±14.03	33.26±19.27	29.03±49.32	0.00	0.00	n.s.
IL-7	14.22±8.48	17.75±15.17	14.15±25.18	0.04	n.s.	n.s.
IL-8	384±631.1	266.57±101.02	115.2±294.7	n.s.	0.00	n.s.
IL-9	55.1±256.25	44.26±51.15	38.38±49.77	n.s.	n.s.	n.s.
IP-10	896.86±593.22	2846.8±3302.56	2942.12±4730.45	0.00	0.00	n.s.
MCP-1	94.56±58	195.1±254.9	147.82±316.37	0.00	0.03	n.s.
MIP-1α	13.06±17.51	13.83±13.53	13.95±18.49	n.s.	n.s.	n.s.
MIP-1β	390.9±306.6	288.97±469.51	217.26±163.84	n.s.	0.00	n.s.
PDGF-BB	4755.18±2061.99	3439.5±1664.07	2444.96±1781.99	0.00	0.00	n.s.
RANTES	57375.59±63290.39	18611.57±11435.01	11487.39±7910.35	0.00	0.00	n.s.
TNF-α	41.93±25.22	117.71±190.76	93.23±237.51	0.00	0.01	n.s.
VEGF	180.75±131.74	249.31±252.37	143.99±125.08	0.01	n.s.	n.s.

a: Mean±SD, pg/ml; b: SLE LN$^-$ vs. NC; c: SLE LN$^+$ vs. NC; d: SLE LN$^+$ vs. SLE LN$^-$; n.s.: not significant
